# School performance gap between non-immigrant and second-generation immigrant children in Sweden—time trends and contributing factors

**DOI:** 10.3389/fpubh.2025.1521387

**Published:** 2025-01-24

**Authors:** Kenta Okuyama, Sara Larsson Lönn, Ardavan M. Khoshnood, Shervin Assari, Jan Sundquist, Kristina Sundquist

**Affiliations:** ^1^Center for Primary Health Care Research, Department of Clinical Sciences Malmö, Lund University, Malmö, Sweden; ^2^Emergency Medicine, Department of Clinical Sciences Malmö, Lund University, Skåne University Hospital, Malmö, Sweden; ^3^Department of Internal Medicine, Charles R Drew University of Medicine and Science, Los Angeles, CA, United States; ^4^Department of Family Medicine, Charles R Drew University of Medicine and Science, Los Angeles, CA, United States; ^5^Department of Urban Public Health, Charles R Drew University of Medicine and Science, Los Angeles, CA, United States; ^6^Marginalization Related Diminished Returns Research Center, Los Angeles, CA, United States; ^7^University Clinic Primary Care Skåne, Region Skåne, Sweden

**Keywords:** gap, immigrant, parental mental disorder, school performance, socioeconomic status

## Abstract

We aimed to investigate the school performance gap and its potential trend from 2010 to 2020 in non-immigrant and second-generation immigrant children in Sweden, whether parental mental disorders and low socioeconomic status contribute to this gap and its trends, and whether the effects of these factors differ by immigration status. We used multiple Swedish population registers, including 829,787 children born 1994–2004. We examined the school performance gap and its trends by the interaction between immigration status and year with linear mixed models. We assessed whether parental mental disorders and socioeconomic status contributed to this gap and its trends, and whether their effects on school performance differ by immigration status. The existing gap was explained by parental mental disorders in addition to parental education and neighborhood socioeconomic status for both males and females. The unadjusted model suggested an increasing trend of the existing gap in school performance by immigration status for both males and females. In the adjusted model, the increasing trend of the gap remained among males and was partially attributed to parental education and neighborhood socioeconomic status. The interaction tests showed that the potential effects of these factors on school performance were smaller among second-generation immigrant children. Efforts to reduce the effects of socioeconomic inequalities and parental mental disorders are warranted in addition to extra support for second-generation immigrant children at schools.

## 1 Introduction

School performance in adolescence is critical for further educational attainment, successful integration into the labor market, and physical, mental, and social well-being in adulthood (1–9). In addition, poor school performance could also lead to negative consequences within societies, e.g., increased risks of violent and other crimes starting in late adolescence and young adulthood, and increased healthcare costs (10, 11). That a gap in school performance exists between students is not new information and this has also been established in repeated Program for International Student Assessment (PISA) results from the OECD and a recent review (10, 12). Low-performing students tend to be immigrants, belong to minority groups, have low family socioeconomic status, and/or live in deprived neighborhoods (10).

Research in the US has investigated the school performance gap between minority groups (Black and Hispanic children) and White children. Most studies identified a large gap in school performance by minority status and the gap remained sizable over time (13, 14). In Sweden, a large and persistent gap in school performance between non-immigrant and immigrant students has also been observed (15–18). These studies indicate that contributing factors to gaps associated with immigrant or minority status are primarily related to socioeconomic status such as parental education, parental income, and neighborhood socioeconomic status (14–17, 19–22). A number of studies further investigated if the potential effects of contributing factors on school performance differed by minority status, and found smaller beneficial effects of parental education among Black and Asian children than White children (19, 22, 23). This is defined as marginalization-related diminished returns (MDRs) (19, 22). Other studies found larger effects of low parental education on children’s health outcomes among immigrants (24). These are important to investigate before the development of targeted interventions.

Besides socioeconomic status, previous research indicates that parental mental disorders are associated with poor school performance regardless of immigration status (25–27). A study has also suggested different effects of parental mental disorders on school performance among males and females (28). According to recent studies, 10–20% of children are exposed to parental mental disorders, especially common mental disorders such as depression and anxiety (29, 30). In addition, immigrants are subjected to a higher risk of common mental disorders due to numerous stress factors accompanied by migration (31). Identifying if parental mental disorders are one of the contributing factors to the gap in school performance between non-immigrant and immigrant children could help to develop targeted and supportive interventions.

In Sweden, the share of people with immigrant backgrounds has increased considerably over the last 50 years. Currently, 26.9% of the population has a foreign background, i.e., first-generation or second-generation immigrants (32, 33). Previous studies have shown that first-generation immigrant children perform worse at school than non-immigrant children (15, 17). On the other hand, there is little research investigating the gap in school performance between non-immigrant and second-generation immigrant children. A report from the Swedish National Agency for Education in 2012 showed that second-generation immigrant children on average perform worse in school than non-immigrant children while the gap was suggested to have decreased between 1998 and 2011 (18). However, whether the gap has changed, contributing factors to the gap and its potential trend are unknown. In particular, second-generation immigrant children who have a non-Western background have not been studied despite that they may be more susceptible to future adverse mental health outcomes (34–37).

To the best of our knowledge, no studies have investigated the gap in school performance and its potential trend in non-immigrant and second-generation immigrant children and their contributing specific factors, i.e., parental mental disorders in addition to socioeconomic status, and whether those effects differ by immigration status. Therefore, our study consisted of three major aims: to investigate the gap in school performance and its potential trend over time between non-immigrant and second-generation immigrant children by using longitudinal population data in Sweden; to investigate if parental mental disorders are contributing factors to the gap and its potential trend, in addition to socioeconomic status; and to investigate if the potential effects of contributing factors differ by immigration status.

## 2 Materials and methods

### 2.1 Data

We linked nationwide Swedish registers via the unique 10-digit identification number assigned at birth or immigration to all Swedish residents. The identification number was replaced by a serial number to ensure pseudonymity. The following nationwide population registers were used: the National School Register, containing information on school grades, the Inpatient Register and the Outpatient Register to assess parental mental disorders, the Total Population Register to assess parental separation and to obtain information of country of birth, the Multi-Generation Register to link offspring to their parents, and the Longitudinal Integration Database for Health Insurance and Labor Market Studies to assess parental education, parental income, and neighborhood socioeconomic status. The study was approved by the Swedish Ethical Review Authority (no. 2021–04268).

### 2.2 Study subjects

We included children who were born in Sweden between 1994 and 2004. For the variable immigration status, the children were divided into the following two groups: children who were born in Sweden to two Swedish-born parents, i.e., non-immigrant children, and children who were born in Sweden to two foreign-born (non-Western countries) parents, i.e., second-generation immigrant children. The categorization of Western and non-Western regions was based on geographical and political similarities that have been used in previous studies (38, 39). Children born in Sweden with one Swedish-born and one foreign-born parent or two Western-born parents were excluded.

### 2.3 Outcome

We used school grades at the end of the compulsory school year, i.e., the ninth grade, when children in most cases were 16 years old. We assessed the outcome during 11 years, i.e., between 2010 and 2020. The school grades ranged from 0 to 320, which represent the total scores of 16 subjects (e.g., mathematics, Swedish, and English). We estimated the mean school grades by year on a continuous scale in the analyses. The specific time period for the assessment of the outcome was chosen because we also wanted to include data on parental mental disorders between ages 7–16 years among the study subjects and the year 2001 was the first year when outpatient data on mental disorders were available in the nationwide registers.

### 2.4 Explanatory variables

For parental mental disorders, we focused on the most common mental disorders, i.e., depressive and anxiety disorders, that have been reported to be associated with school performance (25–27, 29, 30). Parental mental disorders were measured when children were at compulsory school and before the year when the children received their final compulsory school grades. We defined children exposed to parental mental disorders if one of their parents had any of the following records by ICD-10 codes in the Inpatient or Outpatient Register: depressive disorders (F32, F33), or anxiety disorders (F40, F41, F43). Inpatient and Outpatient Registers contain the clinical diagnoses from specialist healthcare services rather than the primary healthcare services and more severe cases needing specialist healthcare were therefore included. Children of parents with other mental disorders were excluded from the study as we aimed to focus on the most common mental disorders.

To assess other potential contributing factors to the gap and its trend, we assessed parental education, parental income, parental separation, and neighborhood socioeconomic status. These variables were assessed 1 year before the children started compulsory school (6–7 years old). However, for parental education, we used the highest educational level of either of the parents registered at any time point before the children’s first compulsory school year and categorized into low (compulsory school graduate), medium (high school graduate), and high (university graduate). In immigrant parents, this information was based on either self-report or individual-level reports from the educational and other institutions in Sweden. Parental income was categorized into low (first quartile), medium (second and third quartiles), and high (fourth quartile) based on family disposable income. Parental separation was categorized into no (living together) and yes (not living together or death of one parent). Neighborhood socioeconomic status was categorized into low, medium, and high based on an established neighborhood deprivation index that was derived from the proportion of low education, low income, unemployment, and social welfare recipients in Small Area Market Statistics (SAMS). The detailed procedure for deriving the neighborhood deprivation index can be found elsewhere (40).

### 2.5 Statistical analysis

First, we derived descriptive statistics of all variables by graduation year from 2010 to 2020. This allowed us to assess the comparability of 11 different birth cohorts in terms of demographic and socioeconomic characteristics. Second, we derived descriptive statistics of all variables by year and immigration status. This allowed us to assess if the demographic and socioeconomic characteristics changed differently over time between non-immigrant and second-generation immigrant children.

To investigate the gap in school performance and its trend over time between non-immigrant and second-generation immigrant children, we used a linear mixed model. Specifically, we estimated the mean school grades by year (0 to 320) as a continuous variable and its interaction with immigration status, accounting for familial clustering. Familial clustering was accounted for by including the random effect of siblings having the same biological mothers (25).

To investigate contributing factors to the gap in school performance and its trend, we added parental mental disorders, parental education, parental income, parental separation, and neighborhood socioeconomic status in the model. First, we included these factors one by one (results not shown) together with the interaction term of each factor and immigration status. By including these interaction terms, we also investigated if the potential effects of contributing factors differ between non-immigrant and second-generation immigrant children. In the final model (model 3), we kept parental education and neighborhood socioeconomic status to examine if parental mental disorders are contributing factors to the gap and its trend. As a sensitivity analysis, we ran a model with all variables.

All analyses were done separately according to children’s sex as the associations between contributing factors and school performance has been shown to differ by sex (28).

## 3 Results

Our study included 70,000 to 85,000 individuals graduating every year of whom approximately 10% were second-generation immigrant children (Table 1). The proportion of second-generation immigrant children increased over time from 9.63% in 2010 to 12.83% in 2020. The proportion of children with high parental education, high parental income, and high neighborhood socioeconomic status increased more among non-immigrant children compared to second-generation immigrant children. The proportion of children with low parental income, parental separation, and low neighborhood socioeconomic status decreased among non-immigrant children while the proportion of those increased among second-generation immigrant children. The proportion of children exposed to parental mental disorders increased more among non-immigrant children compared to that in second-generation immigrant children. The changes in the proportions of demographic and socioeconomic characteristics from 2010 to 2020 by immigration status are summarized in the Supplementary Figures S1A–E.

**Table 1:** Descriptive statistics of study subjects by year and immigration status.

		2010	2011	2012	2013	2014	2015	2016	2017	2018	2019	2020
	n	86,634	82,202	75,816	70,752	70,818	69,767	71,160	71,978	74,798	77,320	78,542
Immigration status												
Non-immigrant	n (%)	78,295 (90.37%)	73,397 (89.29%)	67,530 (89.07%)	62,811 (88.78%)	62,544 (88.32%)	61,651 (88.37%)	62,919 (88.42%)	63,531 (88.26%)	65,961 (88.19%)	67,856 (87.76%)	68,463 (87.17%)
Second generation	n (%)	8,339 (9.63%)	8,805 (10.71%)	8,286 (10.93%)	7,941 (11.22%)	8,274 (11.68%)	8,116 (11.63%)	8,241 (11.58%)	8,447 (11.74%)	8,837 (11.81%)	9,464 (12.24%)	10,079 (12.83%)
Children’s sex												
Non-immigrant	Females	38,735 (49.47%)	35,748 (48.7%)	33,108 (49.03%)	30,606 (48.73%)	30,227 (48.33%)	29,970 (48.61%)	30,441 (48.38%)	30,659 (48.26%)	32,127 (48.71%)	32,982 (48.61%)	33,406 (48.79%)
	Males	39,560 (50.53%)	37,649 (51.3%)	34,422 (50.97%)	32,205 (51.27%)	32,317 (51.67%)	31,681 (51.39%)	32,478 (51.62%)	32,872 (51.74%)	33,834 (51.29%)	34,874 (51.39%)	35,057 (51.21%)
Second generation	Females	4,148 (49.74%)	4,229 (48.03%)	4,098 (49.46%)	3,931 (49.5%)	4,083 (49.35%)	4,045 (49.84%)	3,999 (48.53%)	4,150 (49.13%)	4,408 (49.88%)	4,672 (49.37%)	4,857 (48.19%)
	Males	4,191 (50.26%)	4,576 (51.97%)	4,188 (50.54%)	4,010 (50.5%)	4,191 (50.65%)	4,071 (50.16%)	4,242 (51.47%)	4,297 (50.87%)	4,429 (50.12%)	4,792 (50.63%)	5,222 (51.81%)
Parental education												
Non-immigrant	High	35,258 (45.03%)	33,658 (45.86%)	31,871 (47.2%)	30,826 (49.08%)	31,849 (50.92%)	32,139 (52.13%)	33,656 (53.49%)	35,412 (55.74%)	37,659 (57.09%)	40,174 (59.2%)	42,032 (61.39%)
	Medium	41,122 (52.52%)	38,007 (51.78%)	34,251 (50.72%)	30,749 (48.95%)	29,541 (47.23%)	28,373 (46.02%)	28,123 (44.7%)	27,136 (42.71%)	27,343 (41.45%)	26,746 (39.42%)	25,533 (37.29%)
	Low	1915 (2.45%)	1732 (2.36%)	1,408 (2.08%)	1,236 (1.97%)	1,154 (1.85%)	1,139 (1.85%)	1,140 (1.81%)	983 (1.55%)	959 (1.45%)	936 (1.38%)	898 (1.31%)
Second generation	High	2,988 (35.83%)	3,101 (35.22%)	2,878 (34.73%)	2,858 (35.99%)	3,014 (36.43%)	2,962 (36.5%)	3,160 (38.34%)	3,374 (39.94%)	3,612 (40.87%)	3,928 (41.5%)	4,328 (42.94%)
	Medium	3,954 (47.42%)	4,258 (48.36%)	4,087 (49.32%)	3,874 (48.78%)	3,905 (47.2%)	3,846 (47.39%)	3,745 (45.44%)	3,802 (45.01%)	3,923 (44.39%)	4,103 (43.35%)	4,302 (42.68%)
	Low	1,397 (16.75%)	1,446 (16.42%)	1,321 (15.94%)	1,209 (15.22%)	1,355 (16.38%)	1,308 (16.12%)	1,336 (16.21%)	1,271 (15.05%)	1,302 (14.73%)	1,433 (15.14%)	1,449 (14.38%)
Parental income												
Non-immigrant	High	37,343 (47.7%)	35,644 (48.57%)	32,681 (48.4%)	31,094 (49.51%)	32,684 (52.26%)	31,407 (50.95%)	32,926 (52.34%)	33,380 (52.55%)	36,114 (54.76%)	37,903 (55.86%)	38,892 (56.81%)
	Medium	33,874 (43.27%)	31,104 (42.38%)	28,657 (42.44%)	26,120 (41.59%)	24,956 (39.9%)	25,258 (40.97%)	25,290 (40.2%)	25,611 (40.32%)	25,296 (38.35%)	25,545 (37.65%)	25,245 (36.88%)
	Low	7,070 (9.03%)	6,642 (9.05%)	6,186 (9.16%)	5,589 (8.9%)	4,899 (7.83%)	4,978 (8.08%)	4,697 (7.47%)	4,535 (7.14%)	4,543 (6.89%)	4,404 (6.49%)	4,323 (6.31%)
Second generation	High	1,261 (15.13%)	1,399 (15.9%)	1,360 (16.43%)	1,294 (16.31%)	1,386 (16.78%)	1,309 (16.16%)	1,357 (16.49%)	1,385 (16.44%)	1,504 (17.08%)	1,541 (16.34%)	1,699 (16.94%)
	Medium	4,833 (58.01%)	5,045 (57.34%)	4,656 (56.25%)	4,564 (57.54%)	4,850 (58.71%)	4,651 (57.41%)	4,814 (58.5%)	4,683 (55.58%)	4,809 (54.62%)	5,187 (55.01%)	5,544 (55.27%)
	Low	2,238 (26.86%)	2,355 (26.76%)	2,262 (27.33%)	2074 (26.15%)	2025 (24.51%)	2,142 (26.44%)	2058 (25.01%)	2,358 (27.98%)	2,491 (28.29%)	2,701 (28.65%)	2,788 (27.79%)
Parental separation												
Non-immigrant	Yes	13,236 (16.91%)	12,499 (17.03%)	11,354 (16.81%)	10,270 (16.35%)	9,705 (15.52%)	9,406 (15.26%)	9,265 (14.73%)	8,938 (14.07%)	9,075 (13.76%)	9,097 (13.41%)	9,355 (13.66%)
	No	65,059 (83.09%)	60,898 (82.97%)	56,176 (83.19%)	52,541 (83.65%)	52,839 (84.48%)	52,245 (84.74%)	53,654 (85.27%)	54,593 (85.93%)	56,886 (86.24%)	58,759 (86.59%)	59,108 (86.34%)
Second generation	Yes	1824 (21.87%)	2063 (23.43%)	1815 (21.9%)	1794 (22.59%)	1869 (22.59%)	1870 (23.04%)	1799 (21.83%)	1820 (21.55%)	1890 (21.39%)	2,125 (22.45%)	2,248 (22.3%)
	No	6,515 (78.13%)	6,742 (76.57%)	6,471 (78.1%)	6,147 (77.41%)	6,405 (77.41%)	6,246 (76.96%)	6,442 (78.17%)	6,627 (78.45%)	6,947 (78.61%)	7,339 (77.55%)	7,831 (77.7%)
Neighborhood socioeconomic status												
Non-immigrant	High	23,472 (30.42%)	21,434 (29.64%)	19,761 (29.64%)	18,544 (29.91%)	18,904 (30.61%)	18,675 (30.7%)	19,156 (30.8%)	21,054 (33.27%)	22,277 (33.88%)	25,216 (37.28%)	25,802 (37.82%)
	Medium	46,441 (60.19%)	44,441 (61.46%)	41,076 (61.62%)	38,099 (61.45%)	37,563 (60.83%)	37,237 (61.21%)	38,301 (61.58%)	37,508 (59.26%)	37,947 (57.71%)	36,186 (53.5%)	36,407 (53.36%)
	Low	7,247 (9.39%)	6,439 (8.9%)	5,823 (8.74%)	5,357 (8.64%)	5,282 (8.55%)	4,918 (8.08%)	4,736 (7.62%)	4,728 (7.47%)	5,531 (8.41%)	6,232 (9.21%)	6,018 (8.82%)
Second generation	High	573 (7.33%)	550 (6.7%)	499 (6.48%)	474 (6.44%)	514 (6.69%)	487 (6.44%)	510 (6.6%)	667 (7.93%)	699 (7.91%)	839 (8.87%)	899 (8.93%)
	Medium	2,689 (34.38%)	2,871 (34.97%)	2,578 (33.48%)	2,326 (31.62%)	2,297 (29.91%)	2,227 (29.45%)	2,452 (31.75%)	2,464 (29.29%)	2,708 (30.66%)	2,694 (28.49%)	2,811 (27.92%)
	Low	4,559 (58.29%)	4,788 (58.33%)	4,623 (60.04%)	4,555 (61.93%)	4,868 (63.39%)	4,849 (64.11%)	4,760 (61.64%)	5,282 (62.78%)	5,426 (61.43%)	5,923 (62.64%)	6,357 (63.15%)
Parental mental disorders												
Non-immigrant	Yes	7,549 (9.64%)	7,459 (10.16%)	7,236 (10.72%)	6,970 (11.1%)	7,041 (11.26%)	7,258 (11.77%)	7,639 (12.14%)	7,678 (12.09%)	8,200 (12.43%)	8,314 (12.25%)	8,390 (12.25%)
	No	70,746 (90.36%)	65,938 (89.84%)	60,294 (89.28%)	55,841 (88.9%)	55,503 (88.74%)	54,393 (88.23%)	55,280 (87.86%)	55,853 (87.91%)	57,761 (87.57%)	59,542 (87.75%)	60,073 (87.75%)
Second generation	Yes	1,505 (18.05%)	1735 (19.7%)	1,596 (19.26%)	1,618 (20.38%)	1723 (20.82%)	1,687 (20.79%)	1721 (20.88%)	1,682 (19.91%)	1753 (19.84%)	1827 (19.3%)	1833 (18.19%)
	No	6,834 (81.95%)	7,070 (80.3%)	6,690 (80.74%)	6,323 (79.62%)	6,551 (79.18%)	6,429 (79.21%)	6,520 (79.12%)	6,765 (80.09%)	7,084 (80.16%)	7,637 (80.7%)	8,246 (81.81%)

**Figure 1:**
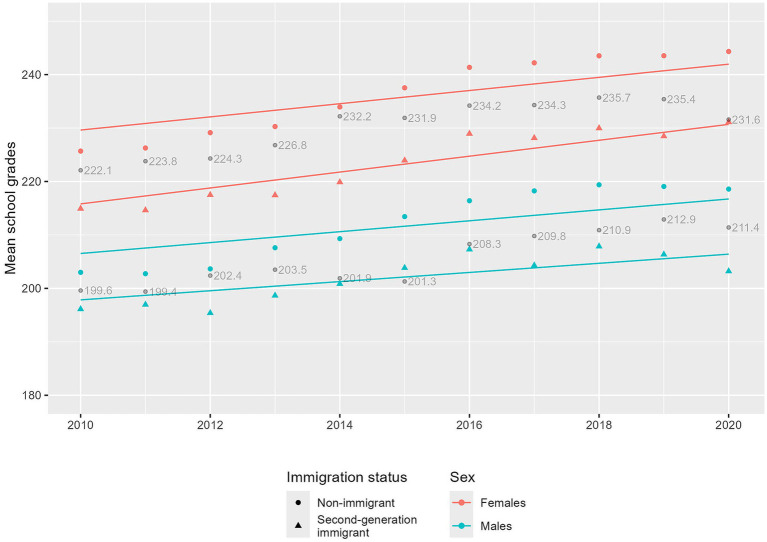
The mean school grades over time by children’s sex and immigration status. Dots represent the observed mean school grades over time. Lines represent the predicted mean school grades from the linear mixed models adjusting for parental education and neighborhood socioeconomic status (Table 2, Model 2). Gray points and numerical values represent mean grades for all children in Sweden available from the National Agency for Education (Skolverket. Statistik. 2024. https://www.skolverket.se/skolutveckling/statistik). **Table:** 

Figure 1 shows that, for both males and females, the observed mean school grades (dots) were higher among non-immigrant than second-generation children across all years. The predicted mean school grades (lines) suggested a slightly decreasing trend of the gap between non-immigrant and second-generation immigrant children for females, i.e., the estimated difference in school grades changed from 7.20 points to 5.90 points between 2010 and 2020, and an increasing trend for males, i.e., the estimated difference in school grades changed from 3.52 to 5.12 between 2010 and 2020.

	Estimated difference in 2010^1^	Estimated difference in 2020^1^
Females	7.20	5.90
Males	3.52	5.12

1 Estimated difference from the predicted lines.

The first model in Table 2 shows that, for both males and females, there was a gap in school performance between non-immigrant and second-generation immigrant children, and this gap increased over time, i.e., school grades increased more among non-immigrant children compared to second-generation immigrant children. Specifically, the gap in school performance increased by 0.20 points every year among females (−0.20, 95%CI: −0.37, −0.02) and 0.61 points among males (−0.61, 95%CI: −0.78, −0.44).

**Table 2:** Results of the linear mixed model for a gap and trend in school grades 2010–2020 by immigration status.

	Coefficient (95% CI)	Coefficient (95% CI)	Coefficient (95% CI)
Females	Model 1^1^	Model 2^2^	Model 3^3^
Intercept	226.36 (226.01, 226.72)	256.25 (255.78, 256.72)	257.32 (256.85, 257.79)
Year (continuous)	1.87 (1.81, 1.93)	1.23 (1.18, 1.29)	1.29 (1.24, 1.35)
Second-generation immigrant (ref Non-immigrant)	−11.14 (−12.25, −10.03)	−7.20 (−9.43, −4.97)	−6.37 (−8.6, −4.14)
Year * Second-generation immigrant	−0.20 (−0.37, −0.02)	0.26 (0.09, 0.42)	0.21 (0.04, 0.37)
Low parental education (ref High)		−75.75 (−77.18, −74.31)	−73.55 (−74.98, −72.12)
Medium parental education (ref High)		−36.88 (−37.28, −36.48)	−36.3 (−36.7, −35.9)
Low neighborhood SES^4^ (ref High)		−24.05 (−24.79, −23.32)	−23.13 (−23.86, −22.39)
Medium neighborhood SES^4^ (ref High)		−10.54 (−10.96, −10.12)	−10.33 (−10.75, −9.91)
Parental mental disorders (ref no)			−16.5 (−17.09, −15.9)
Low parental education * Second-generation immigrant		32.79 (30.59, 35)	30.94 (28.75, 33.14)
Medium parental education * Second-generation immigrant		15.81 (14.54, 17.08)	15.41 (14.15, 16.67)
Low neighborhood SES^4^ * Second-generation immigrant		3.17 (0.97, 5.36)	2.61 (0.42, 4.79)
Medium neighborhood SES^4^ * Second-generation immigrant		−1.96 (−4.15, 0.23)	−1.85 (−4.03, 0.33)
Parental mental disorders * Second-generation immigrant			4.11 (2.64, 5.58)
ICC^5^ family	0.5161	0.4267	0.42
Males			
Intercept	203.25 (202.9, 203.6)	235.09 (234.64, 235.54)	236.04 (235.58, 236.49)
Year (continuous)	1.66 (1.6, 1.72)	1.02 (0.97, 1.07)	1.07 (1.02, 1.13)
Second-generation immigrant (ref Non-immigrant)	−6.10 (−7.18, −5.02)	−3.52 (−5.7, −1.35)	−2.57 (−4.75, −0.39)
Year * Second-generation immigrant	−0.61 (−0.78, −0.44)	−0.16 (−0.32, 0)	−0.21 (−0.37, −0.05)
Low parental education (ref High)		−73.53 (−74.93, −72.14)	−71.65 (−73.04, −70.26)
Medium parental education (ref High)		−37.97 (−38.36, −37.58)	−37.5 (−37.89, −37.11)
Low neighborhood SES^4^ (ref High)		−25.17 (−25.87, −24.46)	−24.28 (−24.99, −23.58)
Medium neighborhood SES^4^ (ref High)		−12.95 (−13.35, −12.54)	−12.73 (−13.13, −12.32)
Parental mental disorders (ref no)			−14.49 (−15.06, −13.92)
Low parental education * Second-generation immigrant		29.5 (27.36, 31.64)	27.89 (25.76, 30.02)
Medium parental education * Second-generation immigrant		17.51 (16.28, 18.74)	17.27 (16.05, 18.5)
Low neighborhood SES^4^ * Second-generation immigrant		3.45 (1.31, 5.59)	2.68 (0.55, 4.81)
Medium neighborhood SES^4^ * Second-generation immigrant		−0.1 (−2.23, 2.03)	−0.27 (−2.39, 1.85)
Parental mental disorders * Second-generation immigrant			3.73 (2.31, 5.15)
ICC^5^ family	0.5337	0.4385	0.4337

1 First model.

2 Second model adjusting for parental education and neighborhood socioeconomic status.

3 Third model adjusting for parental education, neighborhood socioeconomic status, and parental mental disorders.

4 SES, socioeconomic status.

5 ICC, intraclass correlation coefficient.

The second model in Table 2 shows that, among females, the gap in school performance between non-immigrant and second-generation immigrant children was partially explained by differences in parental education and neighborhood socioeconomic status, i.e., the coefficient was attenuated from −11.14 to −7.20. This gap was slightly further explained by adding parental mental disorders in the third model; here, the coefficient was attenuated from −7.20 to −6.37. On the other hand, the increasing trend of the gap was fully explained by parental education and neighborhood socioeconomic status, i.e., the coefficient of the interaction term of year and immigrant status changed from −0.20 to 0.26. In fact, after accounting for parental education and neighborhood socioeconomic status, a decreasing trend of the gap was observed among females, i.e., the gap decreased by 0.26 points every year, as seen in Figure 1. The interaction terms of parental education, neighborhood socioeconomic status, and parental mental disorders by immigration status suggested weaker associations with these factors among second-generation immigrant than non-immigrant children. Among males, similar to females, the gap in school performance between non-immigrant and second-generation immigrant children was partially explained by parental education and neighborhood socioeconomic status, i.e., the coefficient was attenuated from −6.10 to −3.52, and then further attenuated by parental mental disorders, i.e., from −3.52 to −2.57. The increasing trend of the gap was partially explained by parental education and neighborhood socioeconomic status, i.e., the coefficient of the interaction term was attenuated from −0.61 to −0.16, but not further when adding parental mental disorders. Figure 1 visually presents this trend. Parental mental disorders seemed not to contribute to the increase in the gap in school performance probably because parental mental disorders increased more among non-immigrant children than among second-generation immigrant children as seen in Supplementary Figure S1. Similar to females, the interaction terms of parental education, neighborhood socioeconomic status, and parental mental disorders indicated weaker associations with these factors among second-generation immigrant than non-immigrant children.

Supplementary Table S1A shows that, among females, the gap in school performance between non-immigrant and second-generation immigrant children was further explained by parental income and separation, i.e., the coefficient was attenuated from −6.37 to −4.85. The association with parental income was smaller among second-generation immigrant than non-immigrant children. Among males, the gap was also partially and further explained by parental income and separation, i.e., the coefficient was attenuated from −2.57 to −0.51 (Supplementary Table S1B). The increasing trend of the gap was slight but could be further explained by parental income and separation. The association with parental income was smaller among second-generation immigrant than non-immigrant children.

## 4 Discussion

We found that parental education and neighborhood socioeconomic status were the major contributing factors and that parental mental disorder also contributed to the gap in school performance between non-immigrant and second-generation immigrant children for both females and males. That could be due to a persistently higher prevalence of parental mental disorders among second-generation immigrant children as seen in Supplementary Figure S1A. In addition, we identified an increasing trend of the gap in school performance between non-immigrant and second-generation immigrant male children between 2010 and 2020 in Sweden. Differences in parental education and neighborhood socioeconomic status were identified as contributing factors to the increase in this gap. That could be due to the increase of socioeconomic inequality over time, i.e., the proportion of high parental education and high neighborhood socioeconomic status increased more among non-immigrant than second-generation immigrant children as seen in Supplementary Figures S1B,C. We also found weaker associations between school performance and parental education, neighborhood socioeconomic status, and parental mental disorders among second-generation immigrant than non-immigrant children.

Our findings suggested that parental mental disorders were additional contributing factors to the gap in school performances according to immigrant status and this is supported by previous studies indicating that children of parents with mental disorders are less likely to perform as well as other children (25–27), and immigrants being more susceptible to mental disorders (31). In our study population, the prevalence of parental mental disorders was persistently higher among second-generation immigrant children than non-immigrant children. Some previous studies have indicated a lower prevalence of mental disorders among immigrants potentially due to a lower healthcare utilization, especially in those who have arrived more recently (41). Our findings were different possibly because the parents had lived in Sweden for at least 7 years and also had children in the country. Meanwhile, the effects of parental mental disorders on school performance were not as substantial as parental education and neighborhood socioeconomic status. This is in line with previous studies that found socioeconomic status to be one of the most important contributing factors in the gap in school performance between non-immigrant and immigrant or minority children (14–17, 19–21), as well as studies that found parental education and other socioeconomic characteristics to have larger effects on children’s school performance than parental mental disorders (25, 26). Nevertheless, efforts to reduce the risk of mental disorders among immigrants may be helpful in reducing the gap in school performance between non-immigrant and second-generation immigrant children.

As a major finding, we identified an increasing trend of the gap in school performance between non-immigrant and second-generation male immigrant children and that could be attributed to the increasing inequalities of parental education and neighborhood socioeconomic status over time. One previous study in Sweden identified an increasing trend of the gap in school performance between non-immigrant and first-generation immigrant children (15). Another Swedish study found a persistent gap over time in school performance between non-immigrant and immigrant children (17). Both studies identified parental socioeconomic status as the most important factor for the gap implicating a need to improve the labor market and educational attainments for immigrants. A notable and additional novelty of our findings is that the increase in socioeconomic inequalities over time was identified as a contributing factor to the increasing trend of the gap. Importantly, our target study population was second-generation immigrant children, who are expected to continue to be a large part of the population (33). Therefore, societal interventions to improve the socioeconomic opportunities of immigrants are needed to reduce the gap in school performance. Improved socioeconomic opportunities of immigrants would also be effective in reducing the risk of mental disorders since higher socioeconomic status is one of the most important preventive factors in mental disorders (42).

However, given that our findings suggest that the increasing trend of the gap was not fully attributed to socioeconomic status, other factors at the school level and individual level should be considered. With regards to school-level factors, the Swedish school system has been reformed several times since 1990 (43, 44). For example, during the 90s, private tax-funded schools were established, which allowed parents to freely choose between schools. This may have created differences between immigrant and non-immigrant students (18, 45). Segregation of schools by immigrant and socioeconomic status has likely also increased since a free choice of school was introduced (18, 46–50). In general, immigrant students are challenged to perform well at schools with a high concentration of immigrant students and/or students with low familial socioeconomic status (51). As for individual-level factors, more support at school for immigrant children to overcome language barriers may be crucial. In Sweden, many measures are already in place such as providing immigrants with opportunities to learn Swedish upon their arrival, allowing students to take courses in their mother tongue, training teachers to improve their multicultural sensitivity, and providing extra hours of schooling for immigrant students (48). Future studies should investigate whether segregated schools are associated with the gap and whether tailored support for immigrant students could be included in evidence-based policies and practices that are cost-effective.

As for the difference in the estimated effects of parental education and neighborhood socioeconomic status, our findings indicated that these effects were smaller among second-generation immigrant children which corroborates previous studies that found smaller protective effects of parental education on school performance among racial/ethnic minorities, which is one aspect of the MDR (19, 22, 23). This implies that improving the educational opportunities for the parents and the socioeconomic status of the neighborhood may not be as effective for the school performance of second-generation immigrant children compared to that for non-immigrant children. It is possible that residential and school segregation as well as discrimination at educational settings may hinder second-generation immigrant children from performing well at school. In fact, the school performance of second-generation immigrant children was poorer than in non-immigrant children. It is difficult to conclude, however, if our findings of MDRs are caused by structural inequalities in the immigrants. Future studies should investigate factors specific to second-generation immigrant children.

Among females, the decreasing trend of the gap was identified after accounting for parental education and neighborhood socioeconomic status. Although the inequalities in socioeconomic status widened, the proportion of high parental education and high neighborhood socioeconomic status increased among second-generation immigrant children as well. Previous studies have suggested that female children may be affected by family circumstances to a greater extent and therefore, the decreasing trend of the gap might have been observed (25, 28). However, there remains a possibility that the gap decreased in the high socioeconomic groups but not in the low socioeconomic groups. Future studies could conduct more in-depth analysis by sex as well as in different socioeconomic strata.

### 4.1 Limitations

First, we measured children’s school performance based on their school grades rather than their actual knowledge or intellectual abilities. However, using school grades enabled us to minimize selection bias, which is often a problem in exams such as the PISA that can only include a small sample from the population. In addition, the same trend, i.e., immigrant students perform worse than non-immigrant students, has been seen in the PISA and national exams (16–18). Second, our measure of parental mental disorders was subjected to measurement bias. For example, we were unable to include parental mental disorders prior to the children’s first compulsory school year, which may have obscured the effects. Previous research has suggested an influence of parental mental disorders on children’s school performance even when diagnosed in the prenatal period (25). Therefore, our findings need to be interpreted as estimated effects of parental mental disorders diagnosed during the compulsory school years. Third, we were unable to account for factors that could have biased our results, such as school segregation and individual language proficiency. While these factors may have affected the school performance, we did adjust for neighborhood socioeconomic status and parental education that are partly related to school segregation. However, we did not have access to language proficiency in our nationwide registers. Taking individual language proficiency into account to identify their potential effects would be informative for more cost-effective targeted policies and practices.

## 5 Conclusion

In line with existing evidence, societal-level efforts to improve socioeconomic opportunities in immigrant populations are warranted. Support for immigrant families to reduce the risk of mental disorders may help their children to perform better at school. Future studies should investigate cost-effective strategies to reduce the gap in school performance between immigrant and non-immigrant children.

## Data Availability

The datasets presented in this article are not readily available due to legal concerns. Further information regarding the register data can be found in the Swedish National Board of Health and Welfare (https://www.socialstyrelsen.se/en/statistics-and-data/registers/).
