# Supplementary material for: School performance gap between non-immigrant and second-generation immigrant children in Sweden—time trends and contributing factors
Source: Front Public Health. 2025 Jan 24;13:1521387. doi: 10.3389/fpubh.2025.1521387 (PMC11802443; doi:10.3389/fpubh.2025.1521387)
Supplement: Supplementary file 1 [file Data_Sheet_1.docx]

Supplementary Material

# Supplementary Tables

**Table S1a:** The results of linear regression for a gap and its trend in school grades 2010-2020 by immigration status - Females

|  | **Model 3** | **Model 4** |
| --- | --- | --- |
|  | **SES^1^ and PMD^2^ adjusted** | **SES^1^, PMD^2^, Income, Separation adjusted** |
|  | **Coefficient (95% CI)** | **Coefficient (95% CI)** |
| (Intercept) | 257.32 (256.85, 257.79) | 260.87 (260.4, 261.35) |
| Year (continuous) | 1.29 (1.24, 1.35) | 1.21 (1.16, 1.27) |
| Second-generation immigrant  (ref Non-immigrant) | -6.37 (-8.6, -4.14) | -4.85 (-7.2, -2.5) |
| Year * Second-generation immigrant | 0.21 (0.04, 0.37) | 0.29 (0.13, 0.46) |
| Low parental education  (ref High) | -73.55 (-74.98, -72.12) | -67.57 (-68.99, -66.14) |
| Medium parental education (ref High) | -36.3 (-36.7, -35.9) | -33.31 (-33.72, -32.91) |
| Low neighborhood SES**^1^**  (ref High) | -23.13 (-23.86, -22.39) | -17.85 (-18.59, -17.12) |
| Medium neighborhood SES**^1^**  (ref High) | -10.33 (-10.75, -9.91) | -7.88 (-8.3, -7.45) |
| Parental mental disorders  (ref no) | -16.5 (-17.09, -15.9) | -13.82 (-14.41, -13.22) |
| Low parental income  (ref High) |  | -14.67 (-15.5, -13.84) |
| Medium parental income (ref High) |  | -9.78 (-10.19, -9.37) |
| Parental separation  (ref No) |  | -10.76 (-11.36, -10.16) |
| Low parental education * Second-generation immigrant | 30.94 (28.75, 33.14) | 26.78 (24.6, 28.97) |
| Medium parental education *  Second-generation immigrant | 15.41 (14.15, 16.67) | 13.16 (11.91, 14.42) |
| Low neighborhood SES**^1^** * Second-generation immigrant | 2.61 (0.42, 4.79) | 1.34 (-0.88, 3.56) |
| Medium neighborhood SES**^1^** *  Second-generation immigrant | -1.85 (-4.03, 0.33) | -2.14 (-4.33, 0.04) |
| Parental mental disorders *  Second-generation immigrant | 4.11 (2.64, 5.58) | 2.55 (1.09, 4.01) |
| Low parental income *  Second-generation immigrant |  | 3.81 (1.85, 5.78) |
| Medium parental income *  Second-generation immigrant |  | 2.96 (1.41, 4.5) |
| Parental separation *  Second-generation immgirant |  | 0.83 (-0.67, 2.34) |
|  |  |  |
| ICC**^3^** family | 0.42 | 0.4042 |

**^1^**SES – socioeconomic status. **^2^**PMD – parental mental disorders. **^3^**ICC – intraclass correlation coefficient.

**Table S1b:** The results of linear regression for a gap and its trend in school grades 2010-2020 by immigration status - Males

|  | **Model 3** | **Model 4** |
| --- | --- | --- |
|  | **SES^1^ and PMD^2^ adjusted** | **SES^1^, PMD^2^, Income, Separation adjusted** |
|  | **Coefficient (95% CI)** | **Coefficient (95% CI)** |
| (Intercept) | 236.04 (235.58, 236.49) | 239.6 (239.14, 240.07) |
| Year (continuous) | 1.07 (1.02, 1.13) | 1 (0.94, 1.05) |
| Second-generation immgirant  (ref Non-immigrant) | -2.57 (-4.75, -0.39) | -0.51 (-2.81, 1.78) |
| Year * Second-generation immigrant | -0.21 (-0.37, -0.05) | -0.13 (-0.29, 0.02) |
| Low parental education  (ref High) | -71.65 (-73.04, -70.26) | -65.6 (-66.98, -64.22) |
| Medium parental education (ref High) | -37.5 (-37.89, -37.11) | -34.46 (-34.85, -34.07) |
| Low neighborhood SES**^1^**  (ref High) | -24.28 (-24.99, -23.58) | -18.99 (-19.71, -18.28) |
| Medium neighborhood SES**^1^**  (ref High) | -12.73 (-13.13, -12.32) | -10.17 (-10.57, -9.76) |
| Parental mental disorders  (ref no) | -14.49 (-15.06, -13.92) | -11.84 (-12.42, -11.27) |
| Low parental income  (ref High) |  | -15.61 (-16.41, -14.81) |
| Medium parental income  (ref High) |  | -10.09 (-10.49, -9.7) |
| Parental separation  (ref No) |  | -10.27 (-10.85, -9.7) |
| Low parental education * Second-generation immigrant | 27.89 (25.76, 30.02) | 23.52 (21.4, 25.65) |
| Medium parental education *  Second-generation immigrant | 17.27 (16.05, 18.5) | 15.06 (13.84, 16.28) |
| Low neighborhood SES**^1^** * Second-generation immigrant | 2.68 (0.55, 4.81) | 1.34 (-0.82, 3.5) |
| Medium neighborhood SES**^1^** *  Second-generation immigrant | -0.27 (-2.39, 1.85) | -0.7 (-2.82, 1.42) |
| Parental mental disorders *  Second-generation immigrant | 3.73 (2.31, 5.15) | 2.35 (0.94, 3.76) |
| Low parental income *  Second-generation immigrant |  | 5.06 (3.16, 6.96) |
| Medium parental income *  Second-generation immigrant |  | 2.59 (1.1, 4.09) |
| Parental separation *  Second-generation immigrant |  | -1.04 (-2.49, 0.42) |
|  |  |  |
| ICC**^3^** family | 0.4337 | 0.4186 |

**^1^**SES – socioeconomic status. **^2^**PMD – parental mental disorders. **^3^**ICC – intraclass correlation coefficient.

# Supplementary Figures

**Figure S1 a-e:** Proportion of each class of variables over time between non-immigrant and second-generation immigrant children.

a.


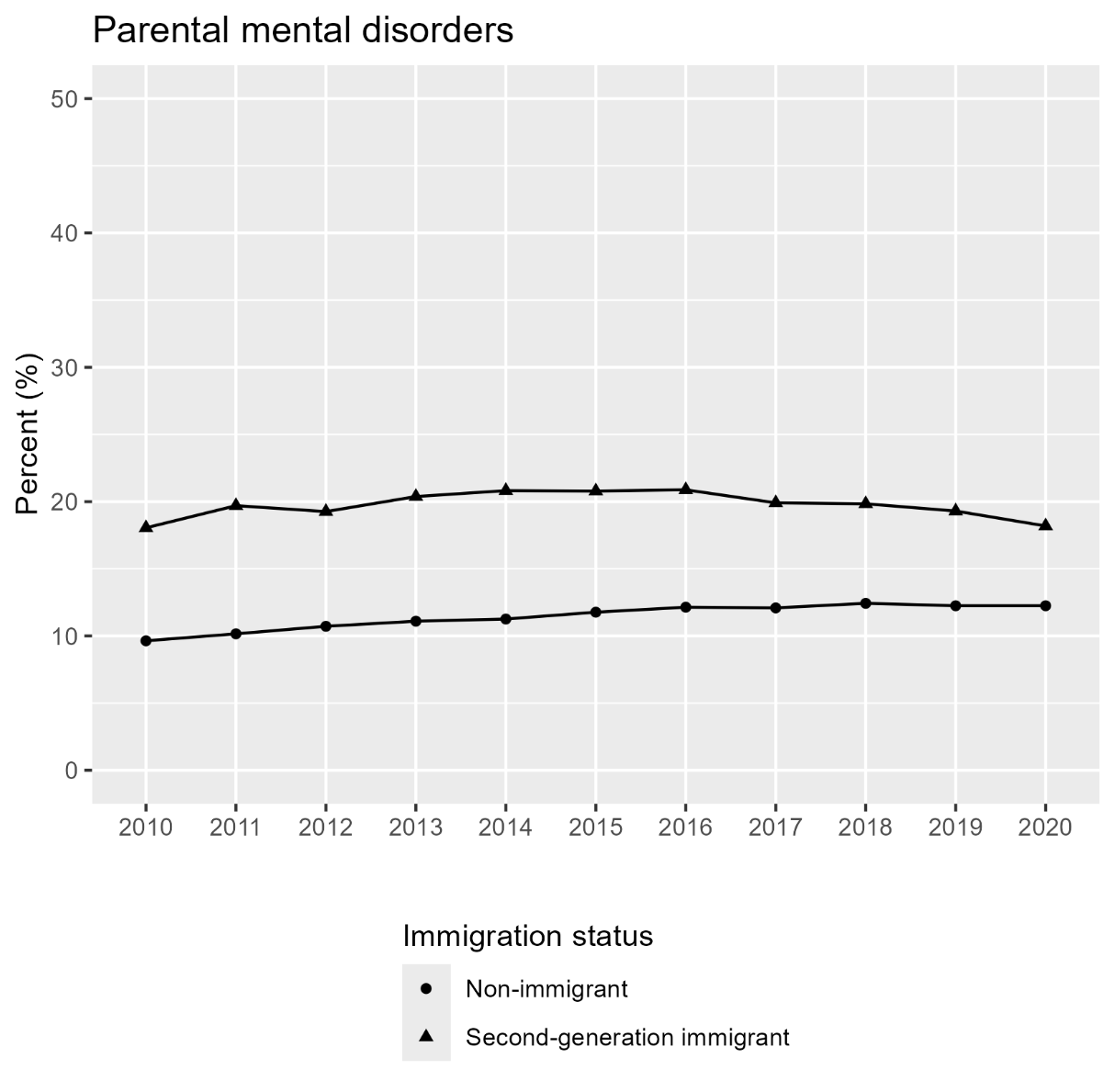


b.


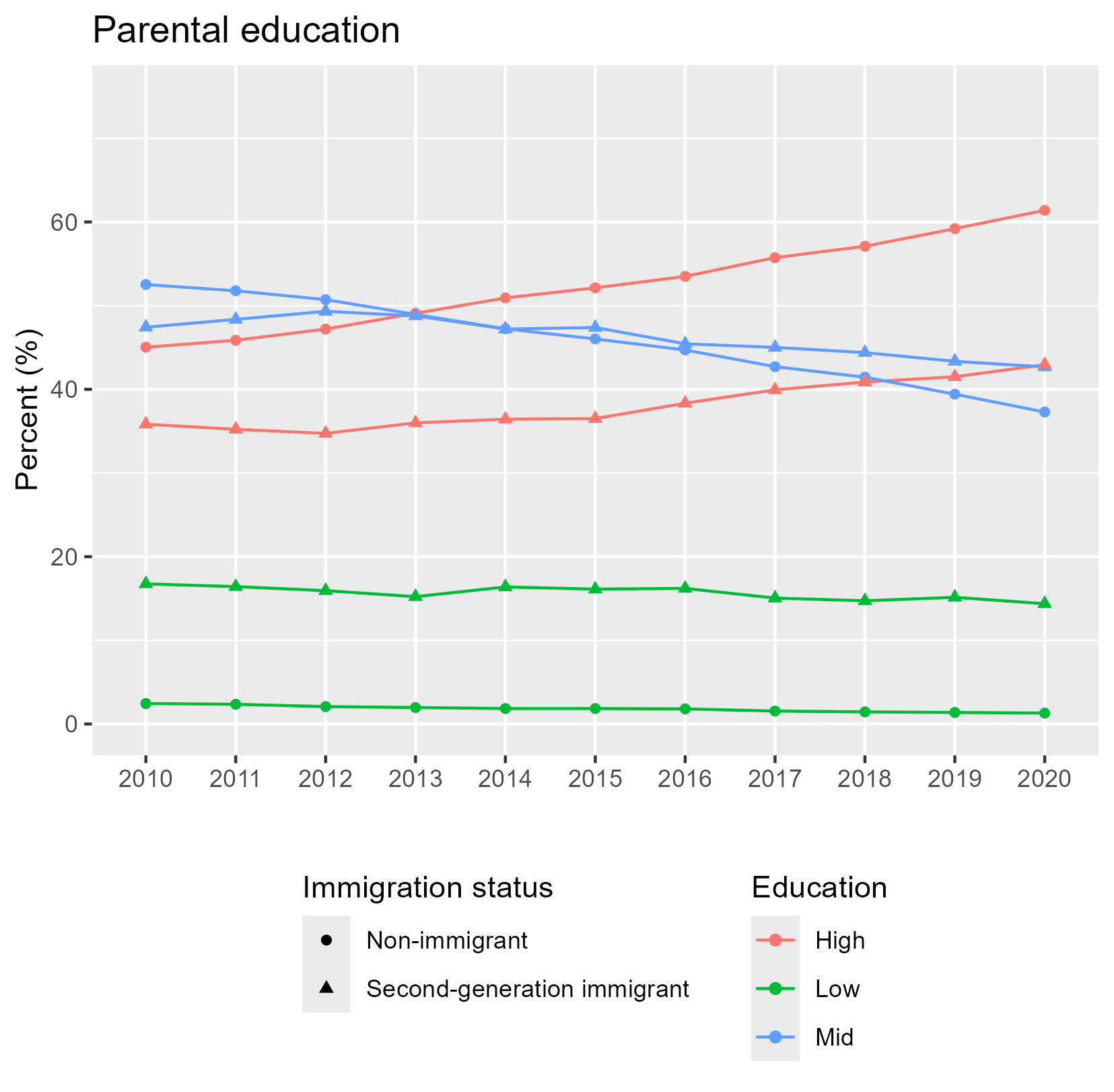


c.


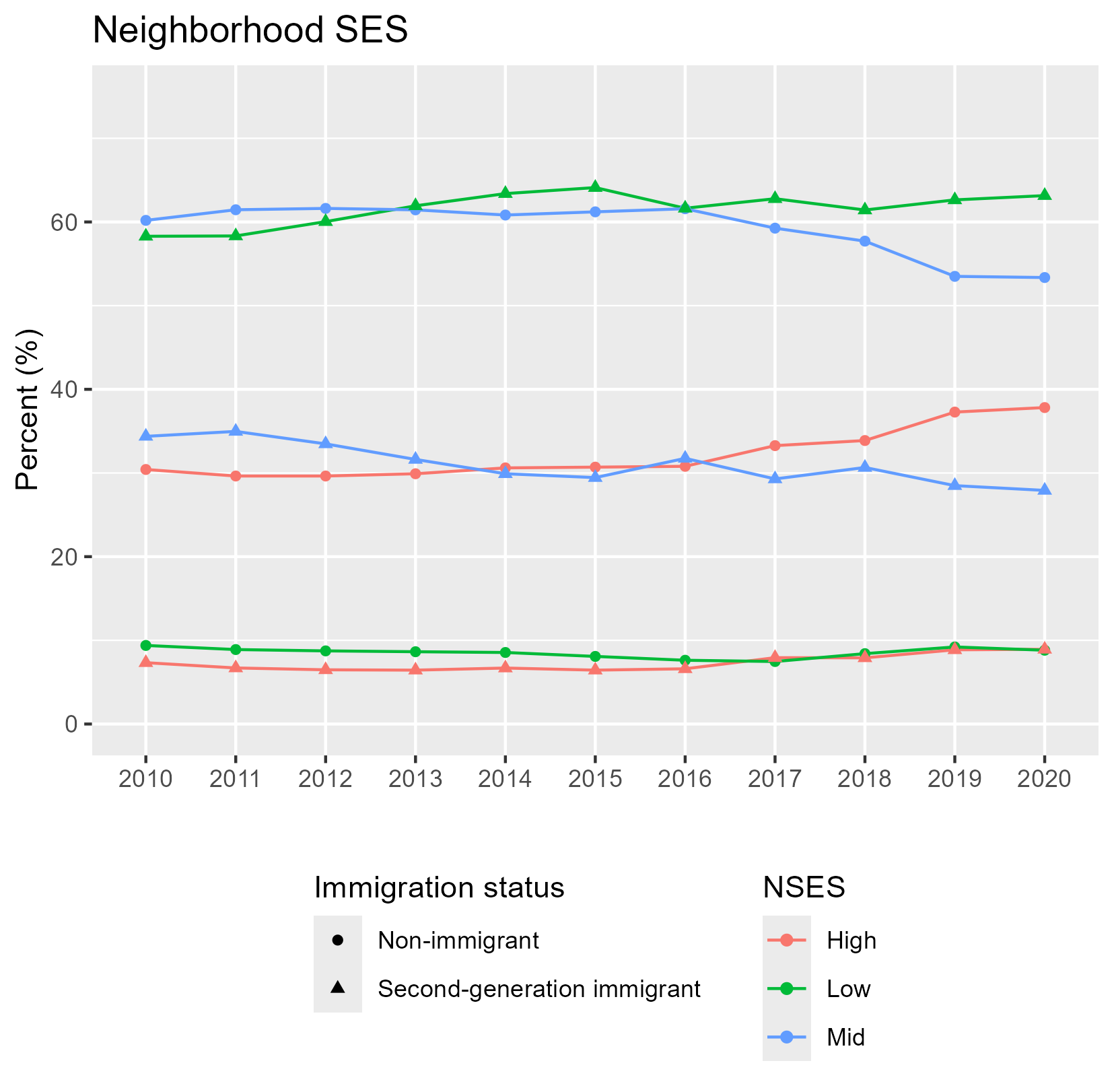


d.


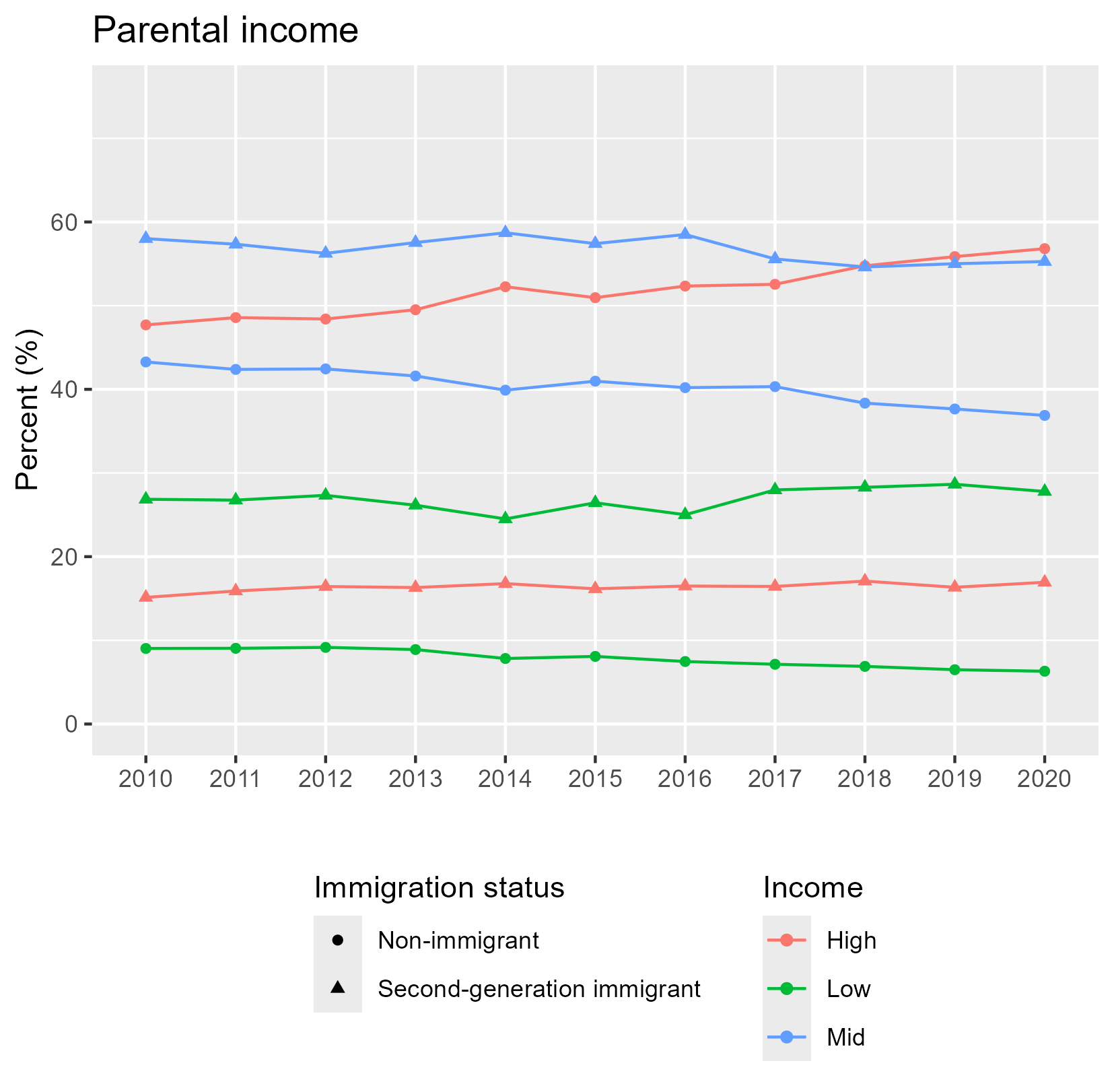


e.

**
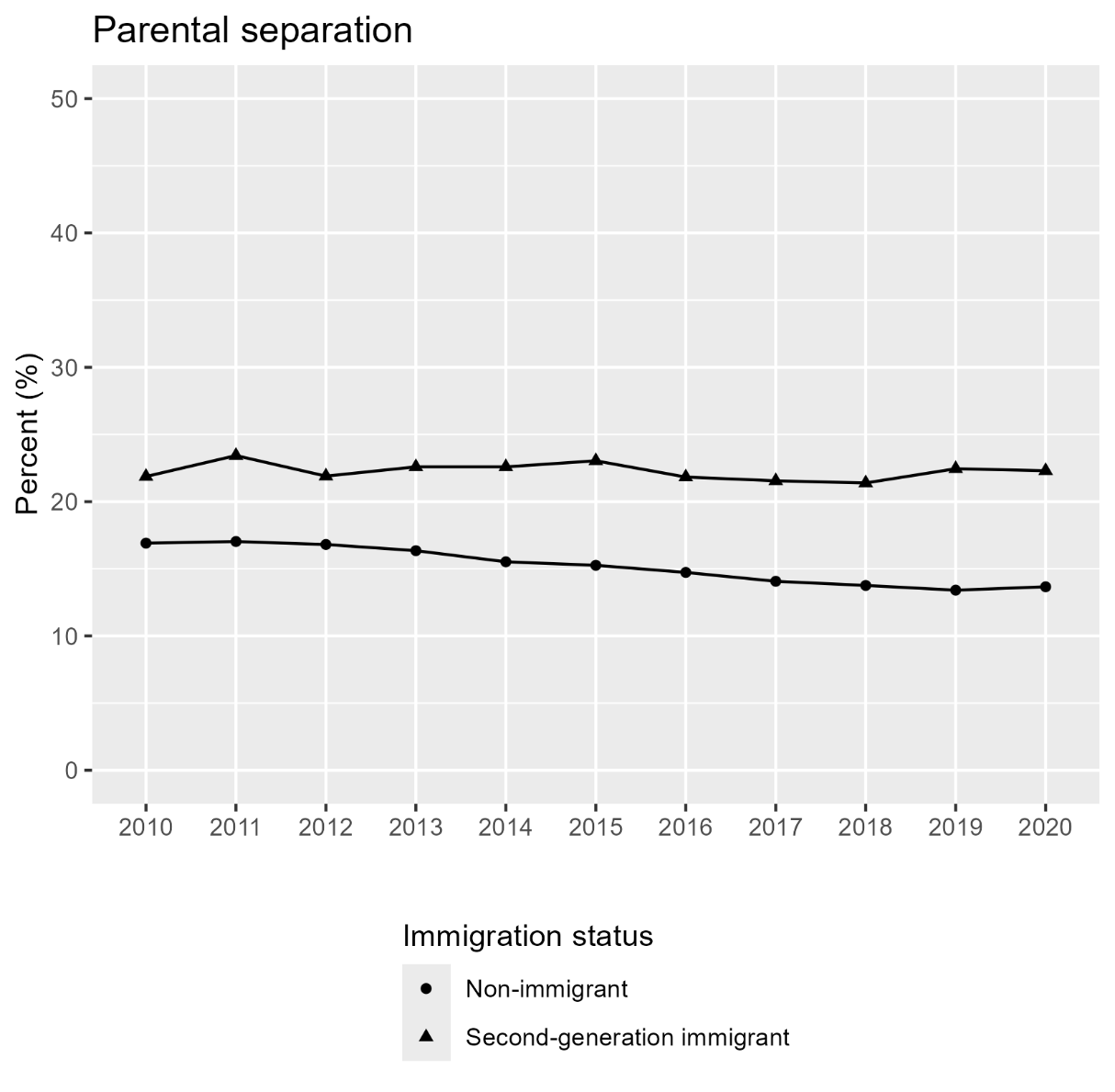
**
